# Endometrioid endometrial cancer “recurring” as high-grade serous adenocarcinoma in the inguinal lymph nodes in a patient with germline MLH1 mutated Lynch syndrome: consequence or coincidence?

**DOI:** 10.1186/s13053-019-0112-x

**Published:** 2019-05-21

**Authors:** Wei Jiang, Tong Gao, Xiang Tao, Menghan Zhu, Liangqing Yao, Weiwei Feng

**Affiliations:** 1 Department of Gynecology, Obstetrics and Gynecology Hospital, Fudan University, 419 Fangxie Road, Shanghai, 200011 China; 2 Shanghai Key Laboratory of Female Reproductive Endocrine Related Diseases, 413 Zhaozhou Road, Shanghai, China; 3 Department of Pathology, Obstetrics and Gynecology Hospital, Fudan University, 419 Fangxie Road, Shanghai, China; 4 Department of Gynecology, Ruijin Hospital of Shanghai Jiao Tong University, 197 Ruijiner Road, Shanghai, China

**Keywords:** Endometrial cancer, Lynch syndrome, Adenocarcinoma, Recurrence, Metastasis

## Abstract

**Background:**

Inguinal metastasis of endometrial cancer (EC) is rare. The aims of the study were to identify whether the inguinal metastatic tumor was originated from EC and to present the management of the disease.

**Methods:**

The clinical data of a case of endometrioid EC “recurring” as serous adenocarcinoma in the inguinal lymph nodes were collected and analyzed. Paired samples of primary and metastatic tumors were used for exome sequencing to determine whether the tumors are same origination and to identify potential gene mutations associated with the relapse.

**Results:**

The patient presented with right inguinal lymphadenopathy and histopathology revealed metastatic serous adenocarcinoma. A germline *MLH1* mutation was identified. A combination of bioinformatical methods and cancer-related gene exome sequencing assay identified that only 17 (0.1%) somatic gene mutations were shared by the primary EC and the metastatic inguinal tumor, suggesting that the metastasis did not originate from the primary EC. Postoperative radiation therapy followed by a combination of chemotherapy were performed. Thirty-four months after that, the patient was doing well without any evidence of recurrence.

**Conclusions:**

This is the first case of metastatic inguinal serous adenocarcinoma in a woman with Lynch syndrome shortly after surgical treatment of stage I endometrioid EC.

**Electronic supplementary material:**

The online version of this article (10.1186/s13053-019-0112-x) contains supplementary material, which is available to authorized users.

## Introduction

Endometrial cancer (EC) is the most common gynecologic malignancy in developed countries [1]. Most patients are diagnosed with localized disease and have an excellent prognosis. However, a high-risk subgroup of women will encounter recurrence and death from this disease [2]. Sites of relapse typically include the vaginal cuff, peritoneum, pelvic and/or para-aortic lymph nodes, and lungs [3]. Anatomically, it is less likely to spread to the superficial or deep inguinal lymph nodes [4].

Approximately 5% of all EC cases are due to a defined inherited cancer syndrome, with Lynch syndrome (LS) being the most commonly associated one [5]. LS is an autosomal dominant cancer syndrome caused by mutations in one of the mismatch repair genes *MLH1, MSH2, MSH6,* and *PMS2*. EC shares with colorectal cancer (CRC) an equal role as a sentinel (i.e., first presenting) malignancy, and the lifetime risk of developing EC in LS patients is approximately 40% [6].

We report the first case of endometrioid endometrial cancer “recurring” as high-grade serous adenocarcinoma in the inguinal lymph nodes in a patient with germline *MLH1* mutated Lynch syndrome. A next-generation cancer-related gene exome sequencing assay for somatic mutations suggested that the metastatic disease did not originate from her primary EC. The patient underwent lymph node dissection, adjuvant radiation therapy and chemotherapy, and after 28 months of follow-up, she experienced complete clinical remission.

## Materials and methods

### H&E and immunohistochemistry

Formalin-fixed and paraffin-embedded sections were reviewed after selection of representative sections for immunohistochemistry. Histological (based on H&E) and immunohistochemical analyses were performed according to standard protocols, as previously described. Monoclonal mouse anti-human ER, PR, Ck7, CK20, MLH1, PMS2, MSH2, MSH6, PTEN, ARID1a, PAX8 and p53 antibodies are from DAKO. In order to find a STIC lesion in the tube or very early ovarian cancer the specimen of the first surgery was serial sectioned of the full specimen and rereviewed by senior pathologists.

### Clinicopathological characteristics

Histopathologic assessment, regarding all slides from the patients in the research, was performed by attending pathologists at our institution. The pathological features of normal and tumor tissues, including primary and metastatic lesions were analyzed. A retrospective extensive review of medical records was performed of patient.

### MLH1 methylation analysis

Analysis of MLH1 promoter methylation was performed on paraffin-embeded primary tumor tissue of the patient. Methylation testing was performed by Methylation-Specific Multiplex Ligation Dependent Probe Amplification (MS-MLPA) using the SALSA MS-MLPA ME011-B1 kit (MRC Holland, Amsterdam, Netherlands). The six probe pairs in the MLH1 promoter (with the respective ligation sites located at − 659, − 518, − 382, − 246, − 13, and + 206 relative to the start codon, LRG_216t1) cover independent regions: regions A to D of the promoter and intron 1. The most important methylation region associated with MLH1 silencing is the C-Deng region, from − 248 to − 178 nt before the transcription site, and the second most important region is the D-Deng region, from − 9 to + 15 nt.

### Genome sequencing

Tumor samples both from primary and metastatic tissues from the patient were available for genome analysis. Each tumor sample was determined as a primary or metastatic by pathologists, with a minimum of 70% of tumor cellularity. Paired samples containing peripheral blood control, primary and metastatic tumors were applied for cancer related gene exome next-generation sequencing to identify potential mutations associated with recurrence, including the Lynch symdrome.

Briefly, total DNA was isolated from frozen peripheral blood and tumor tissue samples using the QIAamp DNA Mini Kit (Qiagen, Hilden, Germany) and the GeneRead DNA FFPE Kit (Qiagen, Hiden, Germany), respectively. DNA concentration was determined using the Qubit dsDNA HS assay kit (Life Technologies) and genomic DNA integrity was assessed by agarose gel electrophoresis. Exome sequencing libraries were prepared using the KAPA Hyper Prep Kit (KAPA Biosystem, Roche), and enrichment was performed with the SeqCapEZ Exomev3.0 Kit (Nimblegen, Roche) following the manufacturer’s instructions. Genomic DNA was sheared to an average size of 200-300 bp using the Covaris M200 sonicator. About 1–1.5μg fragmented genomic DNA was used for end-repairing, A-tailing and adaptor-ligation. Samples were barcoded using illumina indexed adaptors. Size selection was performed before PCR enrichment using Ampure XP beads (Beckman). After library construction, libraries from the same group of samples (based on DNA quality) were pooled together for capture enrichment. Captured libraries were sequenced with the Illumina Hiseq platform using 150 bp paired-end sequencing mode.

## Results

**Fig. 1:**
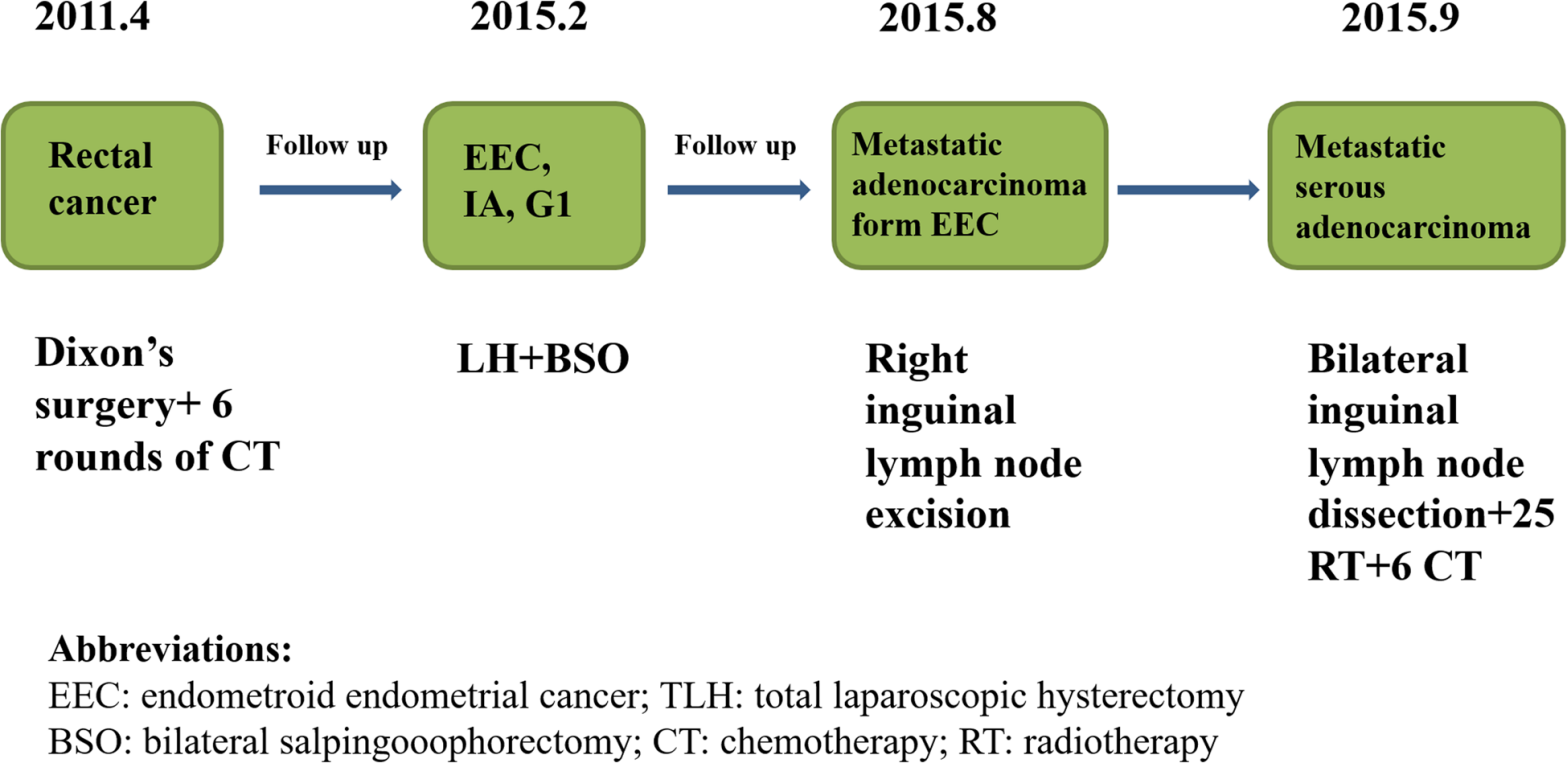
Schematic diagram of disease progression and management

**Fig. 2:**
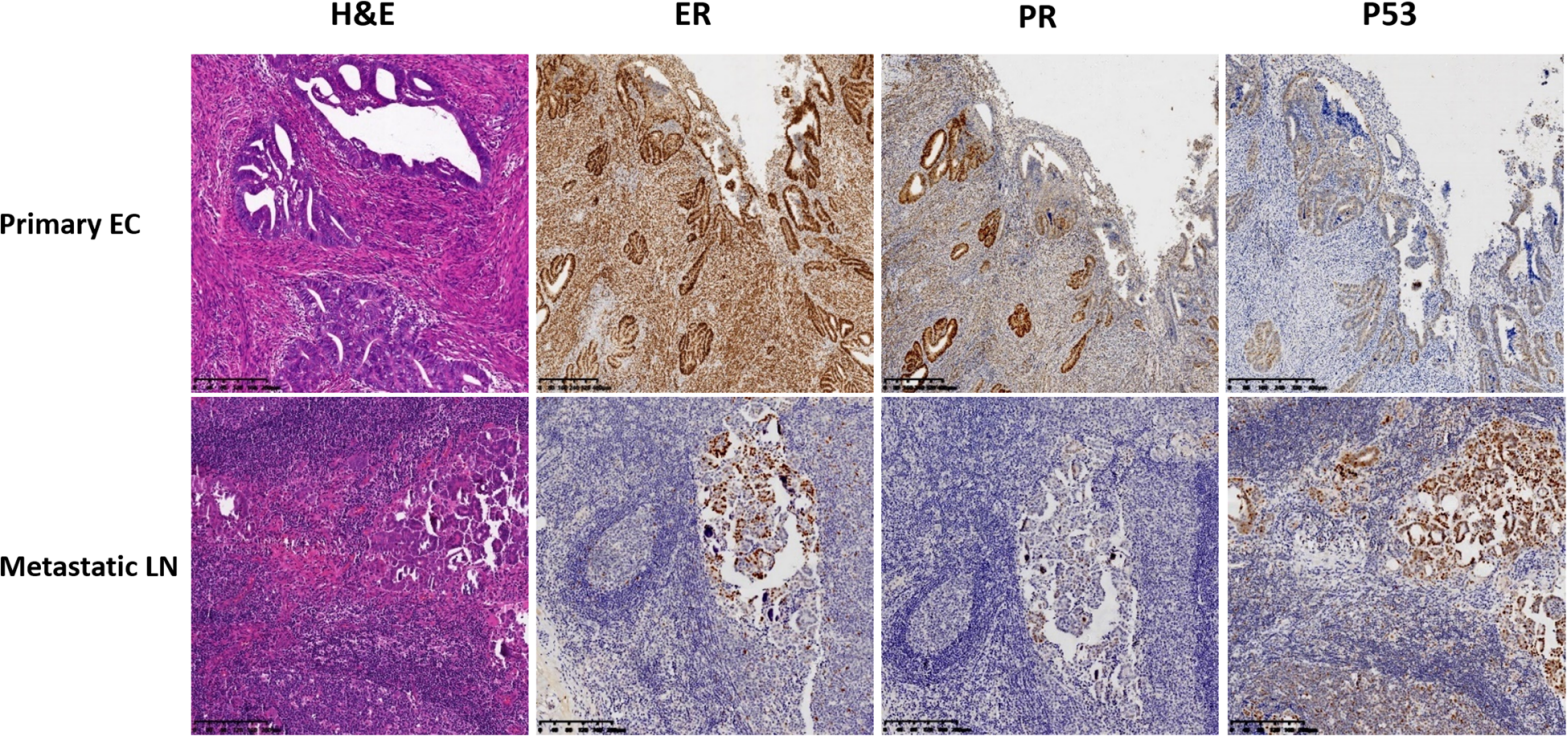
H&E and immunohistochemical staining of primary endometrial cancer and metastatic serous adenocarcinoma in the inguinal lymph node in this patient (original magnification × 10, bar = 200 μm). Monoclonal mouse anti-human ER, PR, and p53 antibodies are from DAKO

The patient presented here was a 54-year-old woman with recurrent FIGO (the International Federation of Gynecology and Obstetrics) stage IA EC, which was treated in the Department of Gynecology at the Obstetrics and Gynecology Hospital of Fudan University in 2015. A summary of the entire disease course and the treatment process is shown in Fig. 1. Her obstetric history included a Cesarean section delivery. She underwent a Dixon surgery and 6 cycles of standard chemotherapy for rectal cancer in 2011. Her menopause was at the age of 50. She had a 3-month history of irregular vaginal bleeding beginning in November 2014, and the diagnosis of EC was made after D&C (dilation and curettage) and histopathology in January 2015. Total laparoscopic hysterectomy and bilateral salpingo-oophorectomy, without lymph node dissection, were performed in February 2015. The frozen sections obtained during surgery were reported to be endometrioid adenocarcinoma within the endometrium. The peritoneal washing fluid was tumor-free. The permanent sections reviewed by gynecologic pathologists revealed grade 1 endometrioid cancer with infiltration of the inner half of the myometrium. The lesion was 0.8 cm in diameter. No malignancies were observed in either the Fallopian tubes or the ovaries. The immunohistochemistry (IHC) results were positive for cytokeratin 7 (CK7), estrogen receptor (ER), progesterone receptor (PR), MSH2, MSH6, PMS2 (weakly positive), PTEN and ARID1a, and they were negative for MLH1 and P53 (Figs. 2 and 4a). The patient recovered uneventfully and was discharged home on the sixth postoperative day with a diagnosis of stage IA G1 EC.

The patient was observed without adjuvant therapy after the initial surgery for EC and received routine follow-up in the outpatient department. Pelvic exam, vaginal smear, transvaginal ultrasound, and Ca125 and HE4 levels were normal at the third and fifth months postoperatively. A small, firm, nonmobile, painless mass was inadvertently palpated in the right groin by the patient at the sixth postoperative month. The serum cancer biomarkers Ca125, Ca153, Ca199, HE4, CEA and AFP were within normal limits. Computed tomography (CT) of her head, neck, chest, abdomen, and pelvis showed no masses or lymphadenopathy. Fluorine-18 fluorodeoxyglucose positron emission tomography-computed tomography (PET-CT) revealed pathologic activity accumulation in only the inguinal nodes and no distant metastasis in September 2015. A right inguinal lymph node excision was performed and metastatic adenocarcinoma in the node was confirmed by histopathological analysis. The diagnosis of recurrent EC in the groin was made. Bilateral superficial inguinal lymph node dissection was performed a few days later. The final report defined the tumor as a metastatic high-grade serous adenocarcinoma of unknown origin that was positive for CK7, ER, paired box gene 8 (PAX8) and p53 and negative for CK20, PR and CDX2 (Fig. 2). The MLH1 and PMS2 were negative in the metastatic serous adenocarcinoma in the inguinal lymph node (Fig. 4b). The patient recovered very well after surgery. Based on the decision of a multidisciplinary team (MDT) at our hospital, postoperative radiation therapy of the bilateral inguinal area was initiated, followed by six 21-day cycles of a combination chemotherapy regime of carboplatin/paclitaxel, which the patient tolerated reasonably well with no major side-effects. Twenty-eight months after bilateral inguinal lymph node dissection, the patient was doing well without any clinical or radiological evidence of locoregional recurrence or distant metastasis.

**Fig. 3:**
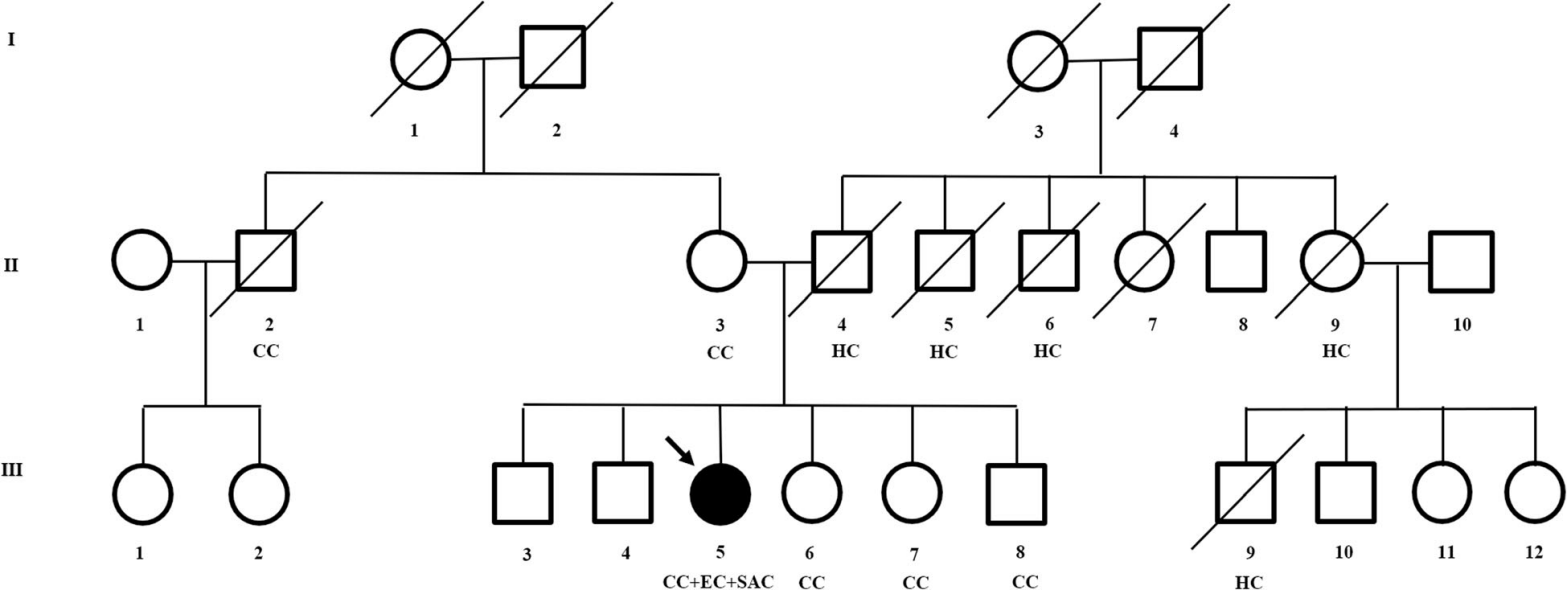
Pedigree structure of the patient’s family. Squares and circles denote males and females, respectively. Roman numerals indicate generations. Arrows indicate the proband (III 5). *CC* colon cancer, *EC* endometrial cancer, *HC* hepatic cancer, *SAC* serous adenocarcinoma

**Fig. 4:**
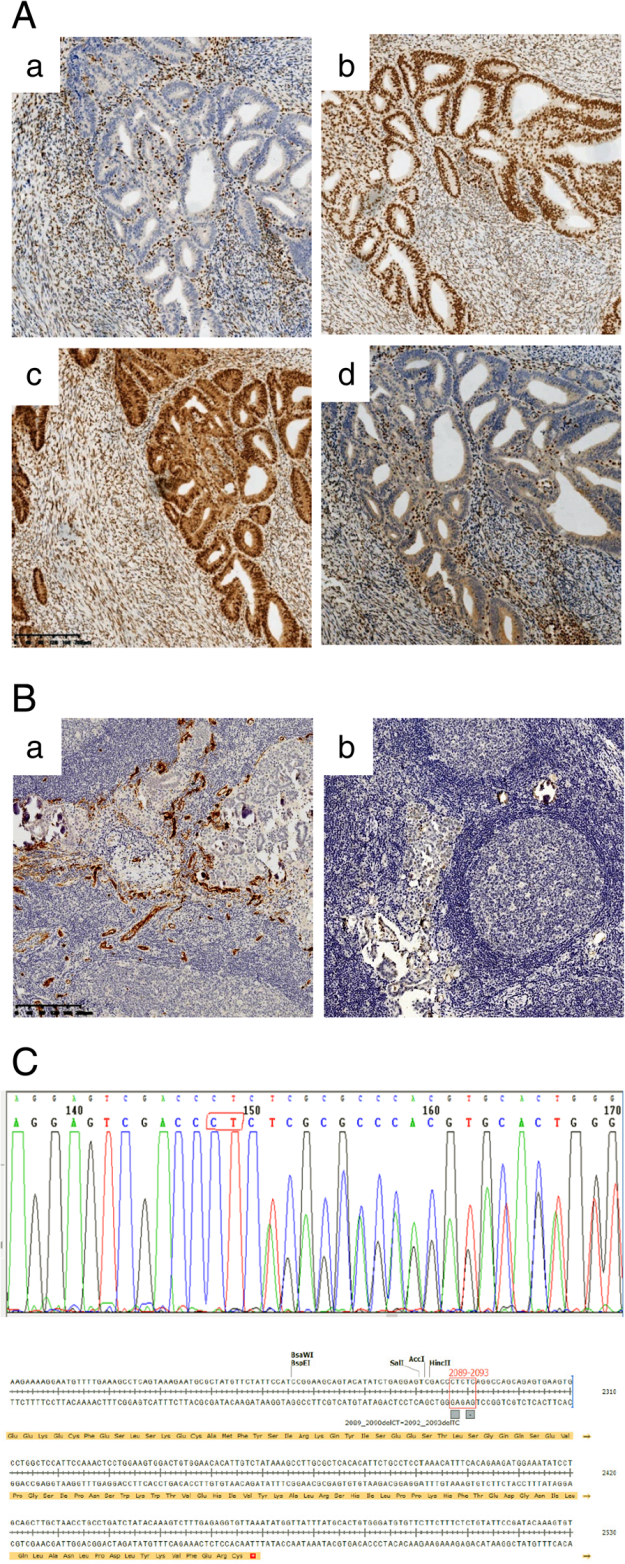
Confirmed Lynch syndrome in the patient. **a**. Immunohistochemical analysis of mismatch repair (MMR) genes for the primary endometrial cancer (original magnification × 10, bar = 200 μm). (a) negative for MLH1. (b) positive for MSH2. (c) positive for MSH6. (d) weakly positive for PMS2. All antibodies are from DAKO. **b**. MMR genes for the metastatic serous adenocarcinoma in the inguinal lymph node (original magnification × 10, bar = 200 μm). (a) negative for MLH1. (b) negative for PMS2. **c**. Germline mutation analysis of MLH1. A germline MLH1 (NM_000249) heterozygote gene mutation (c.2089_2090delCT) was identified in the patient

The patient and her relatives all received genetic counselling to better understand the possible risk factors for EC, to guide future treatments for the patient, and to aid in cancer prevention. We found 5 CRC patients including her siblings, her mother and her mother’s siblings and 5 hepatic cancer patients including her father and her father’s siblings (Fig. 3). Based on the fact that IHC of the primary EC tissues was negative for MLH1 and weekly positive for PMS2 in this patient, MLH1 promoter hypermethylation should be ruled out firstly. We performed MLH1 methylation analysis by MS-MLPA and no constitutional methylation of the MLH1 promoter was detected in the primary tumor of this EC patient. (data not shown). *MLH1* mutation status was then analyzed. Genomic DNA was extracted from the patient’s peripheral blood leukocytes. All *MLH1* coding exons were amplified by polymerase chain reaction and subjected to automatic DNA sequencing. A germline *MLH1* heterozygote gene mutation (NM_000249, c.2089_2090delCT) was identified in the patient (Fig. 4c). The mutation is reported as a pathogenetic mutation in the ClinVar database and is associated with hereditary nonpolyposis colorectal syndrome (HNPCC), also known as Lynch syndrome. The diagnosis of LS was then confirmed according to the revised Amsterdam II criteria.

**Fig. 5:**
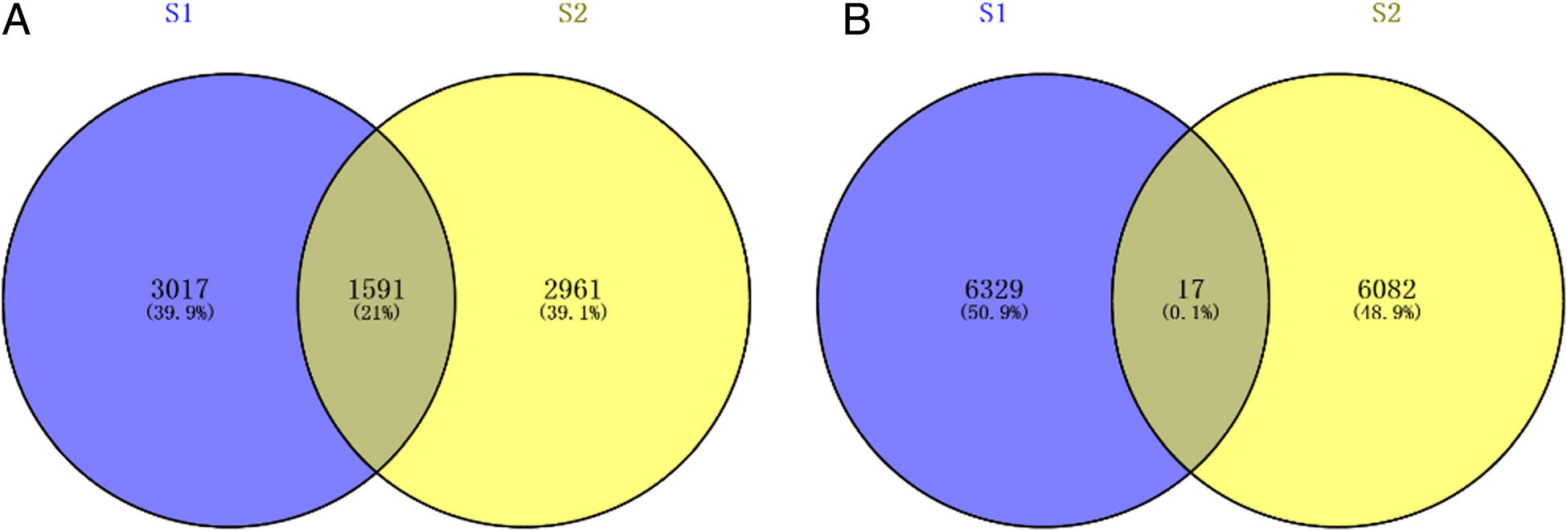
Venn diagram showing somatic mutations in the primary and metastatic tumors. **a**. All gene mutations in the primary and metastatic tumors. **b**. Somatic mutations in the primary and metastatic tumors. In the primary tumors, 6329 filtered somatic mutations were found, and in the metastatic tumors, 6082 mutations were found. Only 17 (0.1%) mutations were shared by both tumors, suggesting that the inguinal lymph node metastasis did not originate from the endometrioid carcinoma

**Fig. 6:**
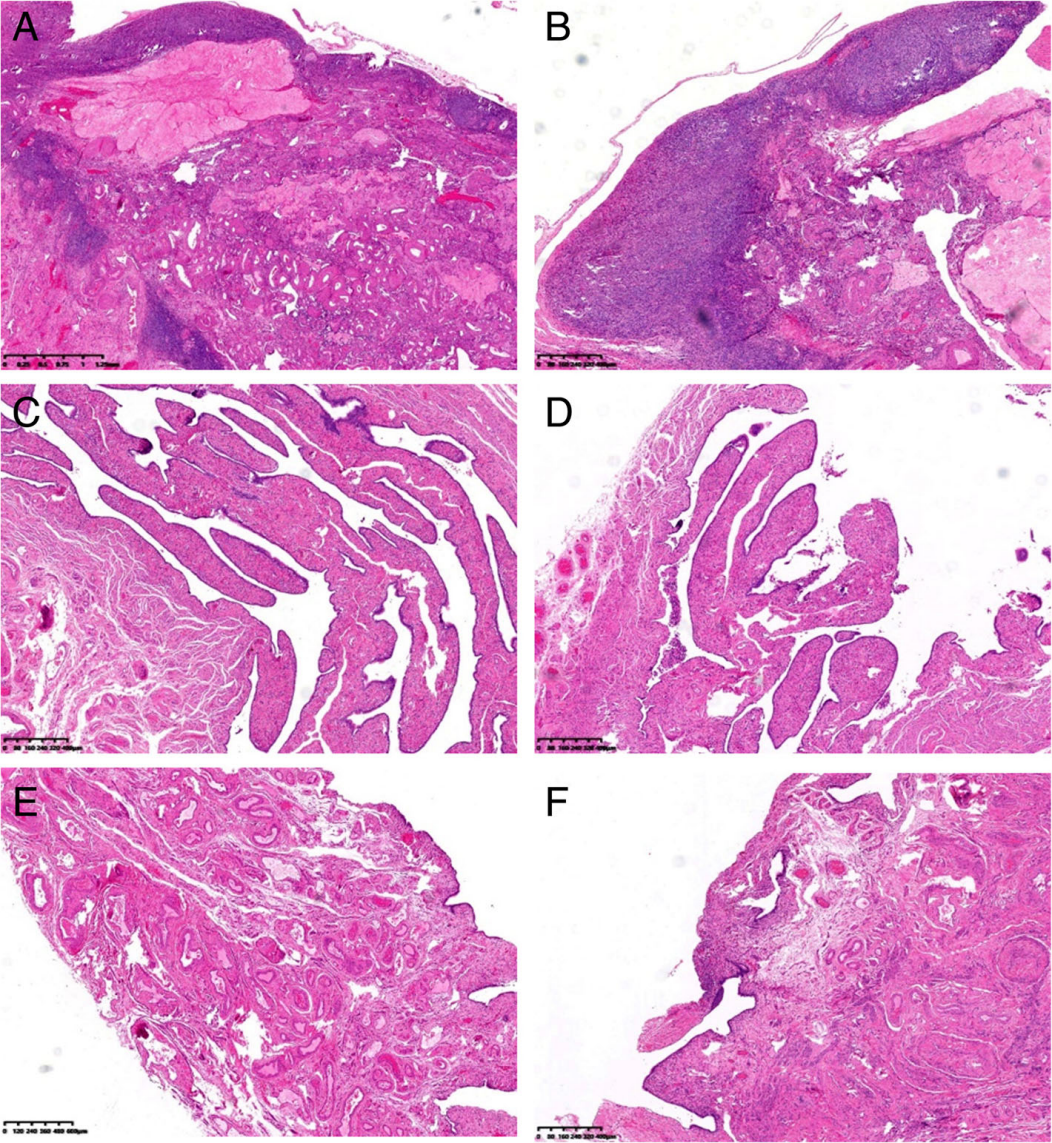
H&E stain of both ovaries and fallopian tubes of the patient showed there was no STIC lesion or early ovarian cancer in the tissues. Serial sectioning of the full specimen from the patient’s initial endometrial cancer surgery was performed and re-evaluated by senior pathologists. From **a** to **f**: left ovary, right ovary, left tube, right tube, left fimbria of tube and right fimbria of tube. (original magnification × 4, bar = 600 μm)

To clarify whether the cancer cells from the high-grade serous adenocarcinoma in the inguinal lymph nodes had the same biological background as the prior EC, that is, to determine whether the metastatic carcinoma in the inguinal lymph nodes originated from the prior EC, we performed a next-generation cancer-related gene exome sequencing assay for somatic mutations in the cancer tissue from the EC and inguinal lymph nodes (Additional files 1 and 2). The results were interpreted by a Venn diagram. In total, 6329 filtered somatic mutations were found in the primary EC, and 6082 mutations were found in the metastatic inguinal tumor. Only 17 (0.1%) mutations were shared by both tumors, suggesting that the inguinal lymph node metastasis did not originate from the EC (Fig. 5). H&E stain of both ovaries and fallopian tubes of the patient showed there was no STIC lesion or early ovarian cancer in the tissues. In order to find a STIC lesion in the tube or very early ovarian cancer, serial sectioning of the full specimen from the patient’s initial endometrial cancer surgery was performed and re-evaluated by senior pathologists. There was no positive finding (Fig. 6). This study was approved by the ethics committee of Obstetrics and Gynecology Hospital of Fudan University. Informed consent was approved by the ethics committee of Obstetrics and Gynecology Hospital of Fudan University and signed by the patient.

## Discussion

Recurrences of early-stage type I endometrial cancer after therapy are unusual, and if they occur, they most often occur locoregionally. Metastatic spread to the lymph nodes occurs in only 4% of cases with grade 1 superficial endometrial cancer [7]. Inguinal lymph node involvement is rare and only a few cases have been reported [4, 8–10]. To our knowledge, this is the first case of metastatic high-grade serous carcinoma of the groin, which was first diagnosed as a relapse of EC. Eventually the possibility of recurrence of EC was excluded in this patient with LS characterized by an *MLH1* mutation.

Determination of the origin of tumor cells is challenging in the absence of an identifiable primary site. Knowledge of the origin of tumor cells helps researchers to better understand carcinogenesis and has implications for diagnosing, classifying, treating, and preventing malignancies. We first speculated that the recurrent serous carcinoma was related to an unrecognized carcinoma in the uterine tissue or adnexa; however, we failed to find any evidence of serous-type malignant cells or epithelial atypia after careful review of all the specimens, including samples from the endometrium, cervix, Fallopian tubes and ovaries, from the patient’s initial surgery. Some might theorize that the metastatic carcinoma could have directly originated from the primary EC and differentiated into a high-grade serous carcinoma in the inguinal lymph nodes. As technology has improved and more sophisticated molecular techniques have developed, new ways of determining the possible origins of cancer cells have also developed [11]. We have successfully identified several patients with lung metastasis from primary low-risk FIGO stage I ECs by using a combination of bioinformatical methods and a next-generation gene exome sequencing assay for somatic mutations in primary and metastatic tumors [12]. In this patient, we used the same methods and confirmed that the metastatic inguinal carcinoma did not spread from the primary EC. We also tried to take her primary rectal cancer tissue for sequencing, unfortunately, since her rectal cancer surgery was performed in a remote small hospital many years ago, the tumor samples were not well preserved, so the cancer tissue could not be obtained for sequencing.

Carcinoma unknown primary accounts for 0.5–15% of all presentations of malignant disease [13]. Of this group of patients, metastatic carcinoma presenting in inguinal nodes from an unknown primary accounts for only 1–3.5% [14]. In 30% of all patients no primary tumor is identified, even after postmortem examination. In our case, as the ovaries, tubes and cervix were not involved, the peritoneum would seem to be the most likely primary site. Other possible primary sites of serous adenocarcinoma include the pancreas, the gastrointestinal tract, the urinary tract and rarely the lungs. Although there were no positive findings on PET-CT examination, it can hardly exclude the primary sites mentioned above. Since the patient had successive rectal cancer, endometrial carcinoma and serous adenocarcinoma, and associated cancers were present in her relatives, and MLH1 protein expression was absent in the EC tissue, LS was highly suspected and finally confirmed by *MLH1* exon sequencing. In women with Lynch syndrome, the lifelong risk of EC is estimated to be 40–60%, and the risk of ovarian cancer is 12%. Based on the fact both MLH1 and PMS2 are negative in her metastatic lymph node tumor, this metastatic tumor must be associated with the Lynch syndrome.

Approximately 60% of patients with carcinoma of unknown primary site do not fit into any specific ‘treatable subset’ and therefore require empiric chemotherapy or radiotherapy [15]. Briasoulis et al. evaluated the efficacy of the carboplatin/paclitaxel combination in patients with carcinoma of unknown primary site, and the overall response rate was 38.7% [16]. Metastatic carcinomas of the inguinal lymph nodes are classified as distant metastases, and they are regarded as a systemic disease. Due to the rarity of this scenario, the impact of inguinal lymph node dissection followed by radiotherapy and systemic chemotherapy on the survival of these patients is not well known; thus, the management is debatable. However, 28 months after bilateral inguinal lymph node dissection, our patient was found to be disease free. In our opinion, the optimal treatment of these rare cases should be discussed by a multidisciplinary team to identify the best possible treatment for each patient, taking into account the possible associated morbidity if a radical surgical approach is taken.

## Conclusions

We report the first case of high-grade metastatic inguinal serous adenocarcinoma in a woman with LS characterized by an *MLH1* mutation shortly after surgical treatment of stage I endometrioid EC. It was ultimately determined by using bioinformatical methods and a next-generation cancer-related gene exome sequencing assay that the metastatic cancer did not originate from the prior EC. Although the primary lesion was not identified, the patient achieved complete clinical remission and a good prognosis through lymph node dissection, followed by local radiotherapy and systemic chemotherapy. This case provides a good example for the management of similar patients in the future.

## Additional files

Additional file 1: The raw data of sequencing results of primary tumors. (XLSX 1198 kb)

Additional file 2: The raw data of sequencing results of metastatic tumors. (XLSX 1156 kb)

## Abbreviations

ARID1a: AT rich interactive domain 1A
CK7: Cytokeratin 7
CRC: Colorectal cancer
CT: Computed tomography
D&C: Silation and curretage
EC: endometrial cancer
ER: estrogen receptor
FIGO: the International Federation of Gynecology and Obstetrics
HNPCC: Hereditary nonpolyposis colorectal syndrome
IHC: Immunohistochemistry
LS: Lynch syndrome
MDT: Multidisciplinary team
PAX8: Paired box gene 8
PET-CT: Positron emission tomography-computed tomography
PR: Progesterone receptor
PTEN: Phosphatase and tensin homolog deleted on chromosome ten
